# Nutraceutical Vegetable Oil Nanoformulations for Prevention and Management of Diseases

**DOI:** 10.3390/nano10061232

**Published:** 2020-06-24

**Authors:** Cristian Vergallo

**Affiliations:** Department of Biological and Environmental Science and Technology (Di.S.Te.B.A.), University of Salento, 73010 Lecce, Italy; cristian.vergallo@unisalento.it

**Keywords:** vegetable oils, nutraceuticals, plant bioactives, nanoformulated delivery systems

## Abstract

The scientific community is becoming increasingly interested in identifying, characterizing, and delivering nutraceuticals, which constitutes a multi-billion-dollar business. These bioactive agents are claimed to exhibit several health benefits, including the prevention and treatment of diseases such as arthritis, cancer, osteoporosis, cataracts, Alzheimer’s, and Huntington’s diseases, heart, brain and metabolic disorders, etc. Nutraceuticals are typically consumed as part of a regular human diet and are usually present within foods, comprising vegetable oil, although at low levels and variable composition. Thus, it is difficult to control the type, amount and frequency of their ingestion by individuals. Nanoformulations about vegetable oil-based bioactive compounds with nutraceutical properties are useful for overcoming these issues, while improving the uptake, absorption, and bioavailability in the body. The purpose of this current study is to review papers on such nanoformulations, particularly those relevant for health benefits and the prevention and management of diseases, as well as bioactives extracted from vegetable oils enhancing the drug effectiveness, retrieved through bibliographic databases by setting a timespan from January 2000 to April 2020 (about 1758 records).

## 1. Introduction

The term ‘nutraceutical’ was coined in 1989 by the physician Stephen DeFelice, chairman of the Foundation for Innovation in Medicine (FIM, Cranford, NJ, U.S.A.), by merging the words ‘nutrition’ and ‘pharmaceutical’. He defined nutraceutical as “[…] a nutritional product—a single entity or combination which includes special diets - that reasonable clinical evidence has shown to have a medical benefit […]” [1]. Over time, this definition has been broadened to make no absolute distinction between foods and drugs. Thus, as nutraceutical is referred to a food (or part of a food) that has medical or health benefits, including the prevention and/or treatment of disease [2]. Nowadays, the term nutraceutical, as commonly used in marketing, is not under any regulation. Nutraceuticals are often referred to as pharma-food, a powerful toolbox administered outside the diet before the drug treatment. It can prevent/treat pathological conditions, such as occurs in patients who are not yet eligible for conventional pharmaceutical therapy [3].

Synthetic drugs are the first treatment option for several diseases. However, pharmaceutical products are strictly regulated and have governmental sanction [4]. The lack of cell specificity of some drugs, like chemotherapeutic agents against cancer cells, contributes to systemic toxicity and adverse effects, which limit the effective dose of the drug administered. Besides, patients have also been concerned because of their high price [5]. Thus, studies which aim to develop alternative therapies in the treatment and prevention of diseases are considerably increasing. Among the alternative therapies, the use of nutraceuticals represents a highly validated approach. Nutraceuticals provide all substances found in food, driving the biological activities that are essential for the human body [6]. Besides, they can prevent or delay several diseases, such as arthritis, cancer, osteoporosis, cataracts, brain disorders, metabolic, cardiovascular, Alzheimer’s, and Huntington’s diseases, etc. Due to their potential nutritional, safety and therapeutic effects, the interest in nutraceuticals is growing rapidly worldwide [7]. The market share of nutraceuticals has been tremendously expanded. The U.S. nutraceutical market was worth approximately 71.73 billion U.S. dollars in 2017 and is forecasted to reach 133.4 billion U.S. dollars by 2025 [8].

**Figure 1:**
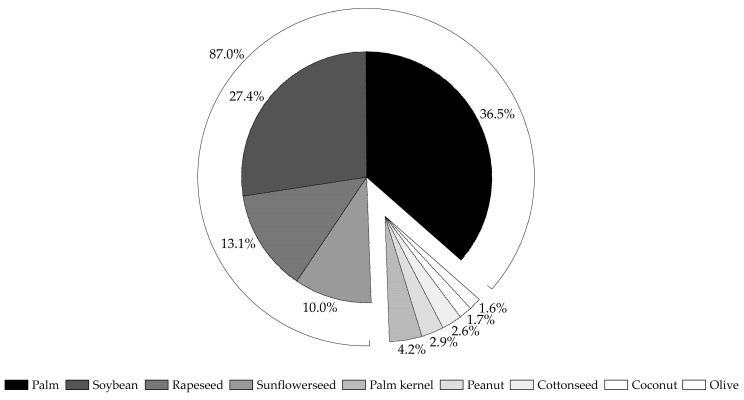
Production (%) of edible vegetable oils worldwide. The production volume of vegetable oil in the 2019/2020 crop year exceeded 200 million metric tons worldwide. Almost 87% of the total world production (180 million metric tons in that period) is represented by the palm (75.69 million metric tons, 36.5%), soybean (56.73 million metric tons, 27.4%), rapeseed (27.04 million metric tons, 13.1%) and sunflower seed (20.65 million metric tons, 10.0%) oils [10].

Vegetable oil is a rich source of nutraceuticals, playing a pivotal role in human health and nutrition, such as carotenoids, lecithin, lignans, oryzanols, phytosterols and phytostanols, policosanol, tocopherols and tocotrienols, triacylglycerols (TAGs) and free fatty acids (FAs) derived from them [6]. As shown in Figure 1, almost 87% of the total world production of edible vegetable oils (180 million metric tons in the 2019/2020 crop year) is represented by four plant oils only, i.e., palm (36.5%), soy (27.4%), rape (13.1%) and sunflower (10.0%). As a result of the high monounsaturation at the crucial 2-position of the oil TAGs, feeding experiments on various animal species and humans have highlighted the beneficial role of fresh palm oil (PO) to health (cholesterol-raising effect), making it as healthy as olive oil [9]. PO had the highest volume of production among the major categories of vegetable oil, with 75.7 million metric tons worldwide. Other important edible plant oils are palm kernel (4.2%), peanut (2.9%), cottonseed (2.6%), coconut (1.7%), and olive (1.6%) oils, which together represent only 27 million metric tons worldwide [10]. Greater amounts of oil are extracted from the seeds of copra (62.5%), palm kernel (44.4%), sun (40.6%), rape (39.1%), peanut (32.7%), soy (18.6%), and cotton (14.4%) [11]. Among the oils extracted from fruit pulp (mesocarp), olive oil and PO are the most marketed. They are contained in the mesocarp in a percentage varying 30–55% (PO) and 38–58% (olive oil) [12].

Challenges about vegetable oil-based nutraceuticals concern issues common to all nutraceuticals, especially when they are administered through the oral route, namely formulation, bioavailability, stability and/or permeation of the bioactive in the gastrointestinal tract (GIT), labile nature, oral absorption and target ability [13,14,15,16]. Researchers are attempting to address these issues by wide spectrum approaches tailored for each specific nutraceutical. The advent of nanotechnology for pharmaceutical applications has opened up a new avenue for enhancing stability, solubility and/or permeation with promising results [15]. Nanocarriers can potentiate the efficacy of plant bioactives by improving their solubility, absorption profile, minimizing dose and side effects [17].

In the present work, a literature review of the past 20 years (January 2000–April 2020) nanoformulations about vegetable oil-based bioactive compounds with nutraceutical properties, i.e., able to exert beneficial effects to health and the prevention and management of diseases, as well as bioactives extracted from vegetable oils used to improve the action of the same drugs, is presented.

## 2. Searching through Bibliographic Databases (Carried out on 4 April 2020 at 1:01 PM)

The following query string was entered into the search-command-line of the database of peer-reviewed literature Scopus (Elsevier):

TITLE-ABS-KEY (((“vegetable” OR “plant” OR “herbal”) AND “oil”) AND ((“profiling” OR “compound*”) AND (“nutr*” OR “nano*”))) AND (LIMIT-TO (PUBYEAR, 2020) OR LIMIT-TO (PUBYEAR, 2019) OR LIMIT-TO (PUBYEAR, 2018) OR LIMIT-TO (PUBYEAR, 2017) OR LIMIT-TO (PUBYEAR, 2016) OR LIMIT-TO (PUBYEAR, 2015) OR LIMIT-TO (PUBYEAR, 2014) OR LIMIT-TO (PUBYEAR, 2013) OR LIMIT-TO (PUBYEAR, 2012) OR LIMIT-TO (PUBYEAR, 2011) OR LIMIT-TO (PUBYEAR, 2010) OR LIMIT-TO (PUBYEAR, 2009) OR LIMIT-TO (PUBYEAR, 2008) OR LIMIT-TO (PUBYEAR, 2007) OR LIMIT-TO (PUBYEAR, 2006) OR LIMIT-TO (PUBYEAR, 2005) OR LIMIT-TO (PUBYEAR, 2004) OR LIMIT-TO (PUBYEAR, 2003) OR LIMIT-TO (PUBYEAR, 2002) OR LIMIT-TO (PUBYEAR, 2001) OR LIMIT-TO (PUBYEAR, 2000)) AND (LIMIT-TO (SUBJAREA, “BIOC”) OR LIMIT-TO (SUBJAREA, “CHEM”) OR LIMIT-TO (SUBJAREA, “MEDI”) OR LIMIT-TO (SUBJAREA, “PHAR”) OR LIMIT-TO (SUBJAREA, “NURS”) OR LIMIT-TO (SUBJAREA, “IMMU”) OR LIMIT-TO (SUBJAREA, “HEAL”) OR LIMIT-TO (SUBJAREA, “NEUR”) OR LIMIT-TO (SUBJAREA, “DENT”)) AND (LIMIT-TO (DOCTYPE, “ar”) OR LIMIT-TO (DOCTYPE, “re”) OR LIMIT-TO (DOCTYPE, “ch”) OR LIMIT-TO (DOCTYPE, “bk”)) AND (LIMIT-TO (LANGUAGE, “English”)) AND (LIMIT-TO (SRCTYPE, “j”) OR LIMIT-TO (SRCTYPE, “b”) OR LIMIT-TO (SRCTYPE, “k”))

The query string was structured to retrieve papers that include, in their title/abstract/keywords, nanoformulations about vegetable oil-based bioactive compounds showing nutraceutical properties, as well as vegetable oil-derived bioactives enhancing the action of the same drugs.

### 2.1. Boolean/Proximity Operators and Wildcard Characters

The following Boolean/Proximity operators and Wildcard characters were adopted to define the query strings:

(1) “” quotation marks. They allow one to find the terms between the quotation marks in the exact order they are specified (exact sentence), avoiding sentences with reversed terms;
(2) () round brackets. They allow one to find the composition of complex search expressions by defining the research priorities;
(3) AND. It allows one to find records in which all the expressions are present simultaneously;
(4) OR. It allows one to find the findings in which there is at least one of the typed terms;
(5) * asterisk. It allows the database to return any word that begins with the root/stem of the term truncated by the asterisk.

### 2.2. General Database Settings

The following general settings were adopted:

(1) English language;
(2) search by topic (title, abstract and keywords);
(3) timespan from January 2000 to April 2020;
(4) document type (articles, reviews, and books);
(5) subject area (chemistry, biology, pharmacology, medicine, and health sciences).

Searches of papers by using other bibliographic databases (PubMed, Web Of Science) were also performed.

### 2.3. Manuscripts Selection

Manuscripts were selected manually by topic (title, abstract and keywords), without considering the impact of them (number of citations or IF/SJR of the journal). Among the papers (articles, reviews, and books) retrieved in the last 20 years (1758 papers, without duplicates), only appropriate findings involving the subject areas of chemistry, biology, pharmacology, medicine, and health sciences were discussed. Herbal bioactive compounds were considered, referring to oils derived from widely diffused and/or cultivate herbaceous plants. Older papers were included in the discussion, just in case they were essential for the description of various topics. In cases where two or more papers discuss the same topic, the most recent, and/or the one containing more data, was considered.

## 3. Profiling of Main Vegetable Oil Bioactive Compounds with Nutraceutical Properties and Relative Affecting Factors

**Figure 2:**
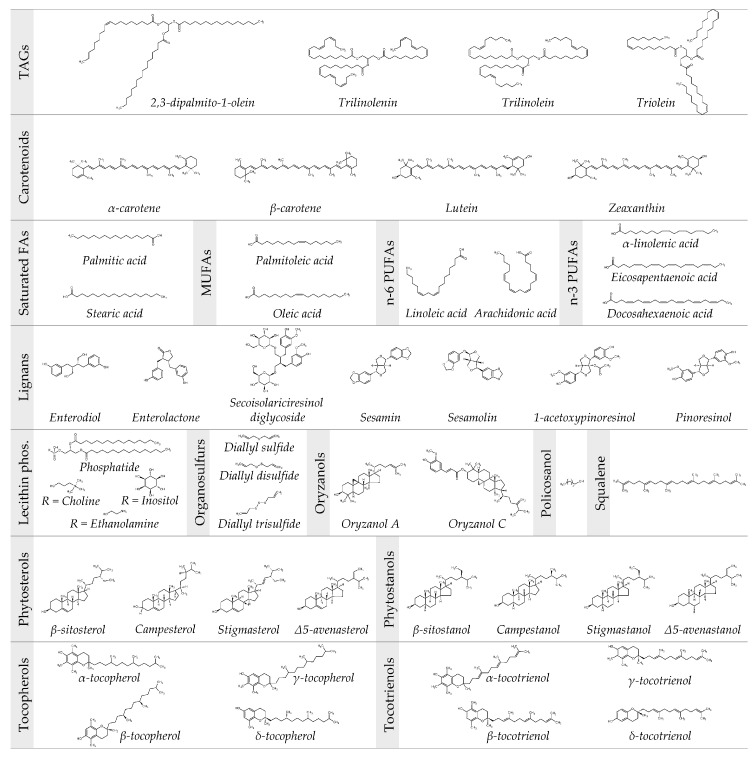
Largest bioactive compounds with nutraceutical properties found in vegetable oils. Lecithin phos. = Lecithin phosphoglycerides.

Among the vegetable oil-based nutraceuticals playing a pivotal role in human health and nutrition, there are TAGs, constituting the major component, and free FAs, carotenoids, lecithin, lignans, oryzanols, phytosterols, phytostanols, policosanol, tocopherols, and tocotrienols, which represent the compounds of the remaining minor fraction [6,18]. Their chemical structures are summarized in Figure 2, whereas a detailed discussion about them is given below.

### 3.1. Major Component

#### 3.1.1. Glycerolipids (mono-, di-, and Triacylglycerols)

Generally, the content of TAGs in a vegetable oil is much higher (95–96%) than that of free FAs, monoacylglycerols (MAGs) and diacylglycerols (DAGs), which are mainly residues obtained from the incomplete biosynthesis of TAGs or products generated as a result of TAGs hydrolysis [19]. TAGs, and FAs derived from them, are evolutionarily considered as excellent energy sources, and therefore they are used either as direct substrates for β-oxidation or stored as a reserve in adipocytes [20]. Some in vivo studies conducted in rats and humans demonstrated that TAGs, especially those bearing saturated FAs in the sn-2 position, have a positive influence on plasma lipoprotein profiles and the reduction of plasma TAG concentrations [21,22]. The TAGs identified in vegetable oil are highly dependent upon the investigation method used to carry out their chemical profiling. TAGs identified in edible vegetable oils extracted from *Arachis hypogaea* L. (*A. hypogaea*, peanut), *Brassica napus* L. (*B. napus*, rapeseed), cottonseed, *Glycine max* L. (*G. max*, soybean), grapeseed, *Helianthus annuus* L. (*H. annuus*, sunflower), linseed, maize, *Olea europaea* L. (*O. europaea*, olive), palm, and by chromatographic, spectrophotometric, and spectroscopic methods, range from 20 to 79, with 6 to 13 different fatty acyl moieties contributing to their molecular structures in grapeseed- and peanut-oil, respectively. The quantitative levels reported range from traces (below 0.1%) to 49.7% (trilinolenin in linseed oil)—54.6% (triolein in olive oil). The highest mean values (30–35%) of TAGs correspond to dipalmitoylolein, trilinolein, trilinolenin, and triolein [19]. Lísa and Holcapek [23] found 264 TAGs after the high-performance liquid chromatography-atmospheric pressure chemical ionization-mass spectrometry (HPLC-APCI-MS) profiling of 26 plant oils relevant in the food, nutrition and cosmetic industries, i.e., blackcurrant, borage, cocoa butter, *Cocos nucifera* L. (*C. nucifera*, coconut), cotton, evening primrose, hazelnut, kukui nut, linseed, olive, palm, peanut, *Persea americana* L. (*P. americana*, avocado) pear, poppy seed, rapeseed, red/white grape seed, redcurrant, safflower, *Sesamum indicum* L. (*S. indicum*, sesame), soybean, sunflower, walnut, wheat germ, and *Zea mays* L. (*Z. mays*, maize). Identified TAGs were composed of 28 FAs with 6–26 carbon atoms and 0–4 double bonds. The equivalent carbon number of all detected TAGs ranged from 32 to 58. Only palmitic-linoleic-palmitic, oleic-oleic-oleic, oleic-oleic-palmitic, palmitic-oleic-palmitic, stearic-oleic-oleic and stearic-oleic-palmitic TAGs were found in all samples. The number of TAGs ranged from simple almond oil and cocoa butter, containing only 25 TAGs, to very complex blackcurrant oil with 77 TAGs, redcurrant oil with 78 TAGs or borage oil with 88 TAGs. TAGs identified in analyzed samples can be divided into three main groups: (i) 5–6 TAGs are found in each sample at the relative concentration level >5%; (ii) from 6 (in almond and hazelnut oils) to 39 (in redcurrant and borage oils) TAGs represent minor constituents with amounts from 0.5% to 5.0%; (iii) trace TAG species (<0.5%) are present in a wide range, from 9 TAGs in cocoa butter oil, up to 51 TAGs in coconut oil.

The melting point is one of the most important physical properties of TAGs affecting the nutraceutical composition of vegetable oil [24]. The melting point depends on the FA composition of TAGs. TAGs with short chain (trilaurin) and unsaturated FAs (triolein) have lower melting points, whereas those with long-chain saturated FAs (tristearin) have higher melting temperatures. Increasing chain length (trilaurin, trimyristin, tripalmitin, and tristearin) leads to raising the melting point. TAGs composed of trans-unsaturated FAs have more elevated melting points than those with FAs having the cis conformation [25]. Interestingly, natural TAGs have somewhat low melting points [26].

### 3.2. Minor Components

#### 3.2.1. Carotenoids

Even though approximately 700 types of natural carotenoids have been identified, the most commonly encountered in foods are α- and β-carotene, lycopene, β-cryptoxanthin, lutein, and zeaxanthin [27]. Based on the chemical structure, carotenoids that exist as pure hydrocarbons are known as carotenes (α- and β-carotene and lycopene), while those that contain oxygen as a functional group in their structure (β-cryptoxanthin, lutein, and zeaxanthin) are referred to as xanthophylls. α-carotene and β-carotene are primarily regarded as precursors of vitamin A. The conversion of β-carotene to vitamin A (retinol) is theoretically higher than α-carotene and β-cryptoxanthin. For instance, one mole of β-carotene yields two moles of retinol, while the other two provitamin-A carotenoids are only as half as active as β-carotene [28]. Notably, β-carotene, lycopene, and derivatives, such as retinoic acid, have been studied for their significant antiproliferative and differentiating activity on cancer cells in experimental models and clinics [29]. *Cyrtostachys renda* L. (*C. renda*, red palm) oil (RPO) contains high concentrations of both α- and β-carotene, ranging from 500 to 800 mg of provitamin A, which are 15 times more elevated than those in carrot when compared on a weight-by-weight basis [30]. However, due to the high percentage of distribution of β-carotene in plant sources, the coexistence of α-carotene and its medicinal values are often less pronounced [28]. Moreover, β-carotene reduces cholesterol biosynthesis in rat liver by inhibiting the enzyme 3-hydroxy-3-methylglutaryl-coenzyme A-reductase (HMGCR) [31]. Dauqan et al. [32] determined spectrophotometerly the concentration of β-carotene in four different vegetable oils, i.e., red palm olein (RPOL), palm olein, maize and coconut oils. Furthermore, β-carotene content (mg/Kg) was found to be as follows: RPOL (542.09) > maize oil (0.91) > palm olein and coconut oil (0.00). Prasanth Kumar and Gopala Krishna [24] dry-fractionated (at 25 °C) crude red palm oil (CRPO) to get crude palm olein (CRPOL, 77%) and crude palm stearin (CRPS, 23%); low and high melting crude palm stearin (14.3% LMCRPS and 8.7% HMCRPS) were separated by the further fractionation of CRPS with acetone. Like Dauqan and co-worker’s data, CRPO contained 514.7 mg/Kg of β-carotene, whereas 82.6%, 16.1%, 12.5% and 3.1% of it was found to be distributed among CRPOL, CRPS, LMCRPS and HMCRPS fractions. PO β-carotene elicits beneficial health effects, sustaining lower total cholesterol levels [9]. Notably, β-carotene contents in oil extracted from rice grain with brown, black and red pericarp are 12.54, 16.22, and 9.98 mg/100 g oil, respectively [33]. Franke et al. [34] quantified by HPLC the carotenoid amount in rapeseed and sunflower marked oils, cold-pressed or refined, and in the oils of rape, sunflower, flax and safflower, including the respective seeds and press cakes from a local oil mill. Lutein content varied between 1.49 (cold-pressed rapeseed marked oil) and 0.03 (sunflower mill oil) mg/100 g fresh matter, with no detectable amounts in refined rapeseed and sunflower marked oils, as well as in mill oils of sunflower/safflower seeds and press cakes. In all oil samples, the content of zeaxanthin was negligible, with slightly higher values found only in the cold-pressed sunflower marked oil (0.09 mg/100 g fresh matter). Pumpkin seed oil is produced in some Central European countries, such as Slovenia, Hungary and Austria, and is considered a preventive agent for different pathologies, particularly prostate diseases. Other than liposoluble vitamins (vitamin E), these properties are related to its high content of lutein and zeaxanthin [35]. Interestingly, in vitro and in vivo studies have provided evidence about the additive and synergistic interaction of carotenoids with various dietary bioactive food components, including E and C vitamins, phenolics, n-3 polyunsaturated fatty acids (PUFAs) and lipoic acid, in preventing oxidative stress, cancer and cardiovascular diseases [36].

#### 3.2.2. Fatty Acids (saturated, mono- and poly-unsaturated)

FAs are classified into: (i) FAs with no unsaturation (e.g., palmitic and stearic acids), (ii) monounsaturated FAs (MUFAs), containing one double bond, and (iii) PUFAs, with more than one double bond. Important MUFAs are the palmitoleic (16:1) and oleic (18:1) acids [37]. It has been found that palmitic and stearic acids, as well as MUFAs, may reduce low-density lipoprotein (LDL) cholesterol, which is a well-known risk factor for coronary heart disease [38,39]. PUFAs include (i) essential FAs (EFAs), namely FAs that humans and other animals must ingest to maintain their body in healthy conditions even if cannot synthesize them, such as n-6 PUFAs/ω-6 FAs (e.g., linoleic and arachidonic acids), and (ii) n-3 PUFAs/ω-3 FAs (e.g., α-linolenic, eicosapentaenoic, and docosahexaenoic acids), based on the distance of the first double bond from the carbon belonging to the methyl end [37]. EFAs are considered as nutraceuticals. Several research studies have documented their pivotal role in many biochemical pathways involved in the concentration of lipoproteins, fluidity of biological membranes, activity of membrane enzymes and receptors, eicosanoids production, blood pressure regulation, and finally, the metabolism of minerals. Therefore, the potential of EFAs to promote healthy activities, with antiatherogenic, antithrombotic, anti-inflammatory, antiarrhythmic, and hypolipidemic effects, as well as to prevent serious diseases, such as cancer, cardiovascular diseases, osteoporosis and diabetes, is considerable [40,41,42,43,44]. The HPLC-APCI-MS profiling of 26 plant oils (see Section 3.1.1) has highlighted how they are almost exclusively constituted by FAs with 16 (palmitic and palmitoleic acids—C_16_) and 18 (stearic, oleic, linoleic, linolenic, γ-linolenic and stearidonic acids—C_18_) carbon atoms, with a total sample concentration ranging from 97.01% (PO) to 99.82% (kukui oil). Lower amounts of C_16_ and C_18_ FAs were found in borage (91.86%) and peanut (90.81%) oils, also containing a higher content of FAs with long acyl chains (C_20_ and longer). The lowest concentrations of C_16_ and C_18_ FAs were detected in coconut oil (23.45%), which instead presented a high percentage of short-chain FAs (C_6_:0 to C_14_:0). The most abundant FAs present in all analysed samples were as follows: (i) palmitic acid (C_16_:0) with concentrations from 5.76% (redcurrant oil) to 40.57% (PO); (ii) stearic acid (C_18_:0) from 0.46% (avocado oil) to 34.51% (cocoa butter); (iii) oleic acid (C_18_:1) from 7.66% (evening primrose oil) to 73.85% (olive oil); (iv) linoleic acid (C_18_:2) from 1.89% (cocoa butter) to 73.96% (safflower oil); and (v) arachidic acid (C_20_:0) from 0.03% (coconut oil) to 1.05% (cocoa butter) [23]. High amounts of oleic and linoleic acids have also been found in oil extracted from whole rice grains with brown, black, and red pericarp, ranging between 40.30–40.49 (oleic acid) % and 37.40–41.39 (linoleic acid) % [33], as well as in *Oryza sativa* L. (*O. sativa*, rice bran) oil, with 38.4% and 34.4%, respectively [45]. Orsavova et al. [37] investigated the FA composition of 14 vegetable oils, i.e., safflower, grape, Silybum marianum, hemp, sunflower, wheat germ, pumpkin seed, sesame, rice bran, almond, rapeseed, peanut, olive, and coconut oil, by using gas chromatography (GC). Respectively, n-6 PUFAs/ω-6 FAs were found 79.0, 74.7, 63.3, 62.4, 62.2, 59.7, 54.2, 40.9, 33.1, 22.8, 19.6, 18.2, 16.4, and 1.6, whereas n-6 PUFAs/ω-6 FAs occurred at 0.2, 0.2, 0.9, 0.4, 0.2, 1.2, 0.1, 0.2, 0.5, 0.0, 1.2, 0.0, 1.6, and 0.0, as expressed by the percentages of total FA methyl esters.

#### 3.2.3. Lecithin

Lecithin is a common and commercial term for a naturally occurring mixture of phosphatides (also called phospholipids or, more recently by biochemists, phosphoglycerides) that ranges in color from light tan to dark reddish-brown and inconsistency from fluid to plastic strong. However, in biochemistry and medicine, the name lecithin is exclusively given to the sn-3 phosphatidylcholine. Lecithin is a gum substance that is found in processed vegetable oils and extracted by degumming. Soybean oil is the most significant source of agricultural lecithin, where it is present at levels of about 1–3%. Lecithin is the most valuable by-product of the soybean oil production industry, owing to its multiple uses in the food and industrial goods. The global market for lecithin is estimated to be in the range of 130,000 metric tons per year [46,47]. Natural phospholipids are accessible on a wide scale and reproducible consistency at a lower cost than synthetic phospholipids. They are well approved by the regulatory authorities and are manufactured using fewer chemicals and solvents at higher yields. Thus, to prevent scale-up issues during pharmaceutical growth and production, natural phospholipids excipients instead of synthetic ones should be employed for pharmaceutical formulations wherever feasible [46,48]. Lecithin is used in a vast range of foods, feed, pharmaceutical and technical applications as a natural emulsifier and surface-active agent, to change the properties of viscosity and crystallization [46,47] (see Section 4.1). In addition to soybean oil, lecithin is also derived from sunflower seed and rapeseed oils. Vegetable de-oiled lecithins, as derived from soybean, sunflower seed and rapeseed, have the following phospholipid and FA compositions: 20–22%, 20–26%, and 23–31% of phosphatidylcholine; 16–22%, 4–10%, and 9–15% of phosphatidylethanolamine; 13–16%, 15–19%, and 15–18% of phosphatidylinositol; 5–10%, 2–5%, and 5–10% of phosphatidic acid; <3% of lysophosphatidylethanolamine; 0.1% of myristic acid; 21%, 16%, and 10% of palmitic acid; 4.7%, 5.3%, and 0.8% of stearic acid; 9.9%, 21%, and 49% of vaccenic acid; 57%, 54%, and 31% of linoleic acid; 5.0%, 0.2%, and 4.4% of γ-linolenic acid; 0.1%, 0.3%, and 0.1% of arachidic acid; and 0.4%, 1.5%, and 0.1% of behenic acid [48]. According to the most legislative definition, U.S. Pharmacopeia (USP) claims that “Lecithin is a complex mixture of acetone-insoluble phosphatides, which consist chiefly of phosphatidylcholine, phosphatidylethanolamine, phosphatidylserine, and phosphatidylinositol, combined with various amounts of other substances such as triglycerides, fatty acids, and carbohydrates, as separated from the crude vegetable oil source. It contains not less than 50.0 per cent of acetone-insoluble matter” [49].

The U.S. Food and Drugs Administration (FDA) has regulated under Title 21, part 184, direct food substances affirmed as generally recognized as safe (GRAS). Lecithin is listed in § 184.1400, enzyme-modified lecithin in § 184.1063. De-oiled lecithin is used as a cholesterol-lowering food supplement, in approved pharmaceutical products since 1950. Soy phospholipids are anyhow an excellent source of EFAs [46]. Clinical trials with relatively small groups of individuals showed mostly positive cholesterol-lowering effects, but placebo diets were not always carefully monitored [46]. As resulted from other clinical trials, administering phosphatidylserine exerts positive effects on animals and humans. However, here too, this data should be further confirmed by performing more extensive long-term clinical trials [50].

#### 3.2.4. Lignans

Lignans are a class of secondary plant metabolites that belong to the group of diphenolic compounds derived from the combination of two phenylpropanoid C_6_-C_3_ units at the β and β′ carbon, and can be linked to the additional ether, lactone, or carbon bonds; they have a chemical structure like the 1,4-diarylbutan. They occur in a wide range of plant families and show a lot of biological activities [51]. The lignans enterodiol and enterolactone, as derivates from lignan precursors by intestinal bacteria, are classified as phytoestrogens [52]. Notably present in flaxseed is the essential dietary lignan secoisolariciresinol diglycoside. Its concentration, of approximately 1–4% w/w, is the highest recorded in edible plants [53]. Kezimana et al. [54] have shown that this diglycoside offers several health benefits, including protective effects against cardiovascular diseases, diabetes, cancer, and mental stress. Shi et al. [55] found that the total lignan content ranges from 2.52 to 12.76 and 3.38 to 11.53 mg/g in sesame seed and oil samples, respectively. Black sesame seeds have a higher sesamin (1.98–9.41 mg/g) and sesamolin (1.06–3.35 mg/g) content compared with the other three sesame seed varieties. Black sesame oils had a greater lignan amount than white sesame oils. Sesamin has been reported to possess in vivo hypocholesterolemic and suppressive attributes activities against chemically induced cancer, lipopolysaccharide, and human LDL-cholesterol [56], as well as towards the amyloid-β peptide (Aβ) brain toxicity, which is proposed to be an early event in the pathogenesis of Alzheimer’s disease [57]. Sesamin and sesamolin exert effective neuroprotection against cerebral ischaemia [58]. Moreover, 1-acetoxypinoresinol and pinoresinol are representative lignans found in extra-virgin olive oil [59]. Both have antioxidant activity [59], while 1-acetoxypinoresinol dramatically reduces the expression of the human epidermal growth factor receptor 2 (HER2) and specifically induces apoptotic cell death in cultured HER2-positive breast cancer cells, with marginal effects against HER2-negative cells [60].

#### 3.2.5. Organosulfurs

Essential oils (EOs) are volatile, highly odorant complex mixtures containing about 20–60 components at quite different concentrations, produced as secondary metabolites by aromatic plants. They are typically obtained through steam or hydrodistillation, which was first developed by Arabs in the Middle Ages. For centuries, plant EOs have been used as therapeutic remedies in traditional medicines. A wealth of scientific studies has shown their biological properties, especially antiseptic property, including bactericidal, virucidal and fungicidal activities, although additional medicinal properties have also been reported [61,62]. Approximately 100 organosulfur compounds have been identified in garlic EO from *Allium sativum* or *Allium ursinum*, with diallyl sulphide (1.6–9.5% in *A. sativum* vs. 0.1–0.3% in *A. ursinum*), diallyl disulfide (20.8–29.1% in *A. sativum* vs. 9.9–20.7% in *A. ursinum*), and diallyl trisulfide (16.8–50.4% in *A. sativum* vs. 5.2–19.6% in *A. ursinum*) as the principal compounds [18]. Allyl isothiocyanate was identified as the main component (71.06%) in the mustard (*Sinapis alba*) seed EO [63]. These compounds allow one to prevent cardiovascular diseases, diabetes and cancer, along with antioxidant, antimicrobial and immunomodulatory activities [16,18,63,64]. Research findings have demonstrated that many of these effects can be explained, at least for *Allium sativum* L. (*A. sativum*, garlic), by the organosulfur property to stimulate the production of the vascular gasotransmitter hydrogen sulfide (H_2_S) and enhance the regulation of endothelial nitric oxide (NO), which induces smooth muscle cell relaxation, vasodilation, and blood pressure reduction [65], as well as affecting cytochrome P450 enzymes, P-glycoprotein, multidrug resistance proteins (Mrp1 and Mrp 2) [16], or some pathways involved in the apoptosis (suppression) and survival (enhancement) of cells [66]. Notwithstanding, the use of garlic oil in the pharmaceutical industry is very limited, due to its lipophilic characteristics, volatilization, strong odour and poor stability in the gastrointestinal fluids, which lowers its bioavailability for systemic circulation, especially upon oral administration [16].

#### 3.2.6. Oryzanols

Notably, γ-oryzanol is an aggregate of ferulic acid esters of triterpene alcohols such as cycloartenol (106 mg%, oryzanol A) and 24-methylene cycloartenol (494 mg%, oryzanol C), derived from rice (*Oryza sativa*) bran oil, an unconventional oil introduced onto the Indian market [45]. It has excellent antioxidant properties, reduces the aggregation of blood platelets and menopause symptoms, and increases muscle mass. Moreover, γ-oryzanol is used to treat hyperlipidaemia because it assists in lowering cholesterol and TAG levels [45,67]. Cycloartenol is structurally like cholesterol, and may compete with its binding sites and sequestrate it [45]. Recently, Ziegler et al. [33] found 11.17, 18.48, and 7.92 mg of γ-oryzanol for every gram of oil extracted from rice grains, with brown, black, and red pericarps, respectively.

#### 3.2.7. Phytosterols and Phytostanols

Cholesterol is the sterol of mammalian cells, whereas multiple sterols, or phytosterols, are produced by plants. Plant sterols, although structurally like cholesterol, are not synthesized by humans. More than 100 types of phytosterols and 4000 other types of triterpenes have been found in plants. Phytostanols are the hydrogenated form of phytosterols, from which they differ for the lack of a double bond at the Δ5 position [68]. In humans, both plant sterols and stanols contribute to lowering serum cholesterol levels and some of them may act against some types of cancers [68,69]. Even though the underlying mechanism for their cholesterol-lowering effect has not been completely understood, it has been partially explained by the competitive inhibition of cholesterol absorption from the small intestine [70]. Phytosterols are biosynthetically derived from squalene. Amaranth, olive, palm, rice bran, and wheat germ oils are good sources of squalene [45,71,72]. Since its discovery in 1926, squalene has been found in many plants. As extensively reported by several in vivo and in vitro studies, it exhibits antioxidant, anti-inflammatory, anti-atherosclerotic, and antineoplastic properties. This bioactive compound accumulates in the liver to decrease cholesterol and TAGs, with these actions being exerted via a complex network of changes in gene expression, at both transcriptional and editing levels [73]. Phytosterols are catalogued into three classes, i.e., 4-desmethylsterols, 4-monomethylsterols and 4,4′-dimethylsterols (triterpene alcohols) [69]. Recently, Yang et al. [74] analyzed by gas chromatography-mass spectrometry (GC-MS) the content and composition of phytosterols in different kinds of vegetable oils, including camellia, flaxseed, grapeseed, maize, olive, peanut, peony seed, rapeseed, rice bran, sesame, soybean, sunflower, and walnut oils. Moreover, β-sitosterol, campesterol, stigmasterol, and ∆5-avenasterol were the main phytosterols for all kinds of vegetable oils. As the predominant phytosterols, β-sitosterol and campesterol represent more than 50% of total phytosterol contents except for camellia oil, in which they accounted for 46.69%. Cycloartanol took up a larger proportion of total phytosterols in camellia oil than other oils. Compared with other vegetable oils, rice bran oil had higher amounts of every kind of phytosterol expect brassicasterol, which was found predominantly in the Brassicaceae family [45,74]. Rapeseed oil, as the production of rape, contains a certain concentration of brassicasterol, with a percentage of 15.29%. A small content of brassicasterol is also detected in soybean oil, with a percentage of 3.60%. Besides, soybean oil has a higher amount of stigmasterol. Compared with other vegetable oils, maize oil and rice bran oil have higher stanols (campestanol, cycloartanol, and 24-methylene-cycloartanol) [74].

The major issue about phytosterols/phytostanols is their poor absorption by the human intestine. Their absorption rates vary upon the specific sterol and stanol, with a general range of 0.5–2% for sterols and 0.04–0.2% for stanols [75]. Following the absorption, these compounds are effectively excreted into the bile by the liver. Therefore, plasma concentrations of plant sterols and stanols are low, of the order 0.3–1.0 mg/dL for plant sterols and 0.002–0.012 mg/dL for stanols. These levels are about 500- and 10,000-fold lower than those of cholesterol, respectively, for sterols and stanols [76]. Plant sterols and stanols were used as capsules, syrups, or suspensions, to achieve substantial hypocholesterolemic effects in the 1950s. Since they had poor water solubility and bioavailability, it was difficult to employ them as pharmaceutical agents, and they were consequently abandoned. In the late 1990s, parallel to the development of the functional food concept, a new interest in plant sterols and stanols arose, especially when the esterification of these compounds facilitated their inclusion into some food products [77]. Another point of concern is that phytosterols content is affected by the FA composition of the part of the plant used, cultivar, crop year, production area, degree ripeness and storage time of fruits, and the method of oil extraction [69]. As corresponding saturated counterpart compounds, this is very likely to also happen for phytostanols.

#### 3.2.8. Policosanol

Policosanol is the generic term for a mixture of primary aliphatic alcohols originally isolated from sugarcane (*Saccharum officinarum* L.) and comprising hexacosanol, octacosanol, and triacontanol as its largest compounds. Policosanol can also be extracted from a variety of other natural sources, such as beeswax, rice bran and wheat germ [78]. The investigation of policosanol hypocholesterolaemic efficacy, by a meta-analysis of different clinical trials involving diabetic patients at high risk of cardiovascular disease and elderly patients at high risk of pharmacological interactions, highlighted that it can reduce LDL-cholesterol by around 20%. In particular, octacosanol downregulates HMGCR [79]. In marketed products, it is often associated with a low dose of mevacoline to obtain a synergistic effect [80]. Hexacosanol (0.566%) and octacosanol (0.035%) have been found in olive oil [72], whereas dotriacontanol, triacontanol and tetracosanol are the major policosanol components present in whole maize seed. Maize pericarp has a higher content of policosanol (72.7–110.9 mg/kg) than the endosperm (4.0–16.2 mg/kg) and germ (19.3–37.1 mg/kg) fractions. Maize pericarp policosanol includes mainly triacontanol (33.63–46.29 mg/kg), dotriacontanol (22.31–39.46 mg/kg) and octacosanol (8.13–14.0 mg/kg). In contrast, the maize germ fraction contains mostly dotriacontanol (>50%) and no triacontanol. Triacontanol and hexacosanol are the major components of maize endosperm policosanol. The level of tetracosanol is highest in the germ fraction and lowest in the endosperm fraction [81].

#### 3.2.9. Tocopherols and Tocotrienols

Edible oils are the major natural sources of tocopherols and their correlative tocotrienols, collectively known as tocols [82]. Tocopherols derive from 2-methyl-6-chromanol, with a side chain of three terpene moieties in saturated form. Depending on the number and position of methyl substitution on the chromanol ring, they are separated into four individual compounds, i.e., α, β, γ, and δ. The chemical structural difference between tocopherols and tocotrienols lies in the fact that tocopherols have saturated side chains, whereas tocotrienols have unsaturated isoprenoid side chains with three carbon-carbon double bonds [69,82]. Vitamin E is a collective term referring to these eight structurally related compounds, namely α-, β-, γ-, and δ-tocopherol and α-, β-, γ-, and δ-tocotrienol [83]. The elevated concentration of these phenolic bioactives found in highly unsaturated edible oils is due to their antioxidant action towards MUFAs and PUFAs (see Section 3.2.2) [45,69]. Tocopherols scavenge free radicals and counteract events leading to the cell ageing, inflammation and heart diseases [69], whereas tocotrienols, like β-carotene, inhibit HMGCR, resulting in hypocholesterolemia [45]. Just like nuts, oilseeds have high lipid content, therefore an equally high concentration of tocols. Conversely, tocols’ presence in fruits and vegetables is generally negligible because of their low lipid amount [82]. Furthermore, α-tocopherol is the major tocopherol found in olive [72], almond [84], peanut [85], and sunflower [34,86] oils. Interestingly, α-tocopherol content was also detected to vary according to: (i) processing. A notable difference in α-tocopherol concentration (mg/100 g fresh matter) was found among rapeseed and sunflower marketed oils, depending on whether they were overgone to refining or cold pressing: cold-pressed (63.5–72.2) vs. refined (45.7–60.8) sunflower oil > refined (27.7–29.9) vs. cold-pressed (26.2–27.0) rapeseed oil [34]; (ii) pericarp colour. Moreover, α-tocopherol had the highest concentrations in oil extracted from rice grains with black pericarp (37.48), followed by brown (18.25) and red pericarps (8.89) [33]; (iii) genetic manipulation. Oil from sunflower cultivars obtained by mutagenesis and genetic recombination also has high amounts of β- and δ-tocopherol than α-tocopherol [87]. Notably, β-tocopherol generally co-elutes with γ-tocopherol [82], which is contained in a similar or higher amount than that of α-tocopherol in rice bran [45], canola, camelina, linseed, maize, soybean, and walnut oils [88,89]. Annatto [90], palm [91], and rice bran [45] oils are important sources of tocotrienols. PO contains α, γ, and δ tocotrienol homologues [92]. A HPLC analysis of coconut and maize oils, palm olein, and RPOL, gave the following results: (i) α-tocopherol (mg/Kg): maize oil (254) > palm olein (218) > RPOL (171) > coconut oil (0); (ii) α-tocotrienol (mg/Kg): RPOL (294) > palm olein (289) > maize oil (24) > coconut oil (0); (iii) γ-tocotrienol (mg/Kg): palm olein (395) > RPOL (367) > maize oil (39) > coconut oil (0); (iv) δ-tocotrienol (mg/Kg): RPOL (126) > palm olein (11) > maize and coconut oils (0) [32]. Hornstra [93] first found that PO has an anti-clotting effect, acting as an antithrombotic agent, like the highly unsaturated sunflower seed oil. In the same year, Rand et al. have shown that PO tocopherols and their relative tocotrienols are able either to increase the synthesis of prostacyclin, a prostaglandin member of the eicosanoid family able to inhibit the blood-clotting process, or to decrease the production of thromboxane, another eicosanoid instead involved in the suppression of the same process [94].

Like phytosterols, the tocopherol content is also affected by many factors, including production area, crop year, fruit (cultivar, ripeness degree, and storage time) and method of oil extraction [69,85,95]. Extracting solvent employed is another important factor influencing the tocols’ content in the sub-fractions obtained. For example, with regards to tocopherol extraction from PO, Prasanth Kumar and Gopala Krishna [24] found 535.5, 587.1, and 308.0 mg/Kg of total tocopherols among CRPO, CRPOL, and CRPS, with a variable content between 305.6 (LMCRPS) and 36.2 mg/Kg (HMCRPS) upon further fractionation of CRPS with acetone.

## 4. Nanoformulations Involving Vegetable Oil-Based Nutraceuticals

**Figure 3:**
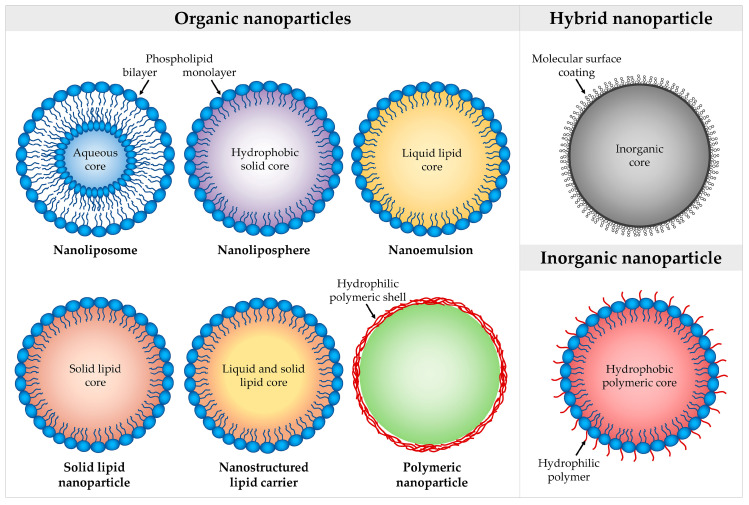
Representative nanocarriers delivering vegetable oil-based nutraceuticals.

Reducing size to nanometric scale gives to compounds distinct and improved properties. Although there is no regulation in the adoption and implementation of legislation and policy and research programs concerning products of nanotechnology, the European Commission invites one to use the following definition of the term ‘nanomaterial’: “ ’Nanomaterial’ means a natural, incidental or manufactured material containing particles, in an unbound state or as an aggregate or as an agglomerate and where, for 50% or more of the particles in the number size distribution, one or more external dimensions is in the size range 1–100 nm. […] In specific cases and where warranted by concerns for the environment, health, safety or competitiveness the number size distribution threshold of 50% may be replaced by a threshold between 1% and 50%” [96]. With this in mind, an in-depth discussion about organic and inorganic nanoparticles (NPs) involving vegetable oil-based nutraceuticals and bioactives extracted from vegetable oils enhancing the drug effectiveness, is presented below, while an overview of their shape is shown in Figure 3.

### 4.1. Organic Nanoparticles

#### 4.1.1. Nanoemulsions

Contrary to conventional drug delivery systems (DDSs), nanoemulsions channel the bioactives to the target site for longer periods and maintain blood-plasma concentration [17], because, such as is extensively documented in the following sections, this improves the solubility and stability, and therefore the low bioavailability of lipophilic compounds. Due to their ability to enhance the therapeutic effect of vegetable oils, nanoemulsions are a promising alternative able to prevent/treat the onset/progression of neoplastic diseases like cancer. This makes them useful for the pharmaceutical industry [97].

##### Oil-in-Water Nanoemulsions

*Ricinus communis* L. (*R. communis*, castor) oil as oily phase, poly(ethylene) glycol 660-12-hydroxystearate and lecithin as surfactants, were used to obtain oil-in-water nanoemulsions able to successfully solubilize quercetin, a natural polyphenol occurring in anti-inflammatory, antibacterial, antioxidant, antiangiogenic, and antitumor activities [98], whereas those prepared with Ultrol^®^ L70/CE200 (surfactants), *P. americana* (avocado) oil, octyl methoxycinnamate and solid particles of TiO_2_ (sunscreens) protected skin from sun exposure [99]. Oil-in-water nanoemulsions containing *Calendula officinalis* L. (*C. officinalis*, calendula) infused black seed oil as the dispersed phase and distilled water as the continuous phase were prepared by using emulsifying agents, including lecithin and Tween^®^ 80, dissolved in the oil phase and aqueous phase, respectively. Corresponding droplet size, PDI and ζ-potential were 54.00–78.29 nm, 0.342–0.370, and −34.00/−39.9 mV. Cytotoxicity, cell-based antioxidant capacity, wound healing and radioprotective activity on monkey-kidney-fibroblast-like cells (Vero) and HaCaT keratinocytes had greater bioactivity than pristine oil, suggesting that these nanoemulsions are a product to include in dermal cosmetics or food supplements with therapeutic efficiency, especially after radio- or chemotherapy [100]. Brownlow et al. [101] nanoemulsified tocotrienol-rich fraction of *C. renda* oil (Tocomin^®^) to yield an optimal nanoemulsion delivery system for dermal photoprotection (droplet size <150 nm, ζ-potential about −30 mV, PDI < 0.25). Prototype Tocomin^®^ nanoemulsion loaded with the antiphotocarcinogenic soy isoflavone genistein showed a slow-release profile in both liquid and cream forms, excellent biocompatibility, and substantial ultraviolet (UV) B protection to cultured subcutaneous L929 fibroblasts. Similarly, *Punica granatum* L. (pomegranate, *P. granatum*) seed oil nanoemulsion entrapping the polyphenol-rich ethyl acetate fraction provided photoprotection against UVB-induced DNA damage in HaCaT cells [102]. Tween^®^ 80 and biodegradable sodium stearoyl lactate (surfactants) were collectively used to fabricate oil-in-water nanoemulsion containing α-tocopherol, benzylisothiocyanate, and curcumin. The prepared emulsion exhibited good stability and cumulative release of these nutraceuticals, acting as a better antioxidant as compared to pure and curcumin encapsulated nanoemulsion. This nanoformulation successfully protected the degradation of curcumin by the impact of ultraviolet (UV) light [97]. Russo et al. [29] developed oil-in-water nanoemulsions by using a carotenoid-enriched extract obtained from the pulp and seeds of *Cucurbita moschata* (*C. moschata*, pumpkin). These nanoemulsions, containing a final carotenoid concentration of 200–400 μg/mL, were not cytotoxic to Caco-2 (colon adenocarcinoma) and SAOs (osteosarcoma) cells. A delay in cell growth of about 40% was also seen. This effect was associated with the activation of a ‘non-protective’ form of autophagy and, in SAOs cells, to the induction of cell differentiation by a mechanism involving 5′ adenosine monophosphate-activated protein kinase (AMPK). A garlic oil blend comprising 30–50% diallyl disulfide, 10–13% diallyl trisulfide, and 5–13% allyl sulfide was nanoemulsified with Tween^®^ 80. Concerning the treatment with unemulsified garlic oil or atorvastatin (statin used to decrease lipid levels and prevent cardiovascular diseases in high-risk patients), the assessment of such garlic oil-based nanoformulations on high-fat diet-fed dyslipidemic or pre-diabetic Wistar rats showed significant ameliorations on abnormal biochemical parameters concerning some urinary/serum lipids and proteins [13,103]. Besides, garlic-oil-based nanoemulsions, when administered to rats, markedly reduced their fat depots [103], with a remarkable attenuation in the mesangial expansion and proliferation, glomerular and tubular basement membrane thickening, and the tubular lipid deposits than its unformulated counterpart or atorvastatin [13]. Compared with normal human foreskin fibroblasts, 36.5 nm stable sour cherry pit oil nanoemulsion significantly decreased the viability of MCF7 cells, reducing the tumor size in a murine breast cancer model too [104].

The volatile nature of EOs, as those isolated from edible plants, presents a major challenge in their incorporation by conventional processing techniques [105]. Nanoemulsification improves the bioavailability of herbal lipophilic agents like EOs, amplifying their antibacterial activity, as seen for *Thymus daenensis* (*T. daenensis*, thymus) EO, which increases its aptitude to disrupt the cell membrane integrity of *Escherichia coli* (*E. coli*) [106] and *Salvia officinalis* (*S. officinalis*, sage) EO against *Haemophilus influenza*, *Moraxella catarrhalis*, *P. aeruginosa*, and *Streptococcus pneumonia*, explained by the ability of the small oil droplets to come into close contact with the microbes (liquid phase) and the slower release of the EO [107]. A nanoemulsion containing the EO of *Rosmarinus officinalis* L. (*R. officinalis*, rosemary), which is mainly composed of limonene, camphor and 1,8-cineole, was more effective (at 600 times lower doses) than unemulsified oil against carrageenan-induced rat paw oedema. Among the major compounds of the EO of rosemary, the camphor molecule exhibited the largest number of interactions with the therapeutic targets related to the inflammatory process, suggesting that it is responsible for the anti-inflammatory and antalgic effects [108]. EOs have proven useful for enhancing the activity of some anticancer chemotherapics, as well as valid agents against tumor growth. The solubility of the antineoplastic drug mitomycin C was obtained by its solubilization in a nanoemulsion, consisting of ginger *Amomum zingiber* L. (*A. zingiber*, ginger) EO. Mitomycin C-loaded ginger EO emulsion endured the nuclear apoptosis of MCF7 breast cancer cells [109]. The nanoemulsion of *Citrus aurantium* L. (*C. aurantium*, bitter orange) bloom EO, containing linalyl acetate, limonene, and α-terpineol as major compounds, affected lung cancer progression. This nanoemulsion was able to onset apoptosis in A549 cells, without inducing a remarkable histopathological alteration of liver and kidney in mice, while it exhibited enhancement in the jejunum morpho-structural architecture and hepatic antioxidant redox potential [110].

##### Self-Nanoemulsions

Self-emulsifying DDS is one type of lipid-based formulation made by an isotropic mixture of natural or synthetic oils, non-ionic surfactants or one/more hydrophilic solvent and co-solvents/surfactant. Self-emulsions are stable DDSs able to increase the drug dissolution, providing a large interfacial area of the dispersion upon oral administration. Mild agitation during gastric mobility induces to form fine emulsions in the GIT, which originate a large interfacial area for drug partitioning between oil and water phases that improves drug solubility and expand absorption. Advantages deriving from the use of these systems include the increased oral bioavailability, reduction in dosage, controlled drug delivery, selective drug targeting, and advanced intestinal lymphatic transport of drugs [111].

Palm kernel oil esters-based nanoemulsions were loaded with 30% *Phyllanthus urinaria* (*P. urinaria*, chamber bitter) ethanolic extract for skin antiaging. These formulations, consisting of *P. urinaria* extract, cetyl alcohol, glyceryl monostearate, palm kernel oil esters, Tween^®^ 80/Span^®^ 80 (surfactants), and a phosphate buffer system at pH 7.4, neutralized reactive oxygen species and counteracted oxidative injury induced by UV radiation, thereby ameliorating skin ageing [112]. Self-nanoemulsions are useful for intensifying the action of some plant bioactives/drugs. With these purposes, self-nanoemulsifying DDSs were prepared to enhance the solubility and dissolution, then bioavailability, of oleanolic acid (also known as oleanic acid; a naturally occurring pentacyclic triterpenoid related to betulinic acid, widely distributed in plants and food such as olives, where it has been isolated as free acid or aglycone of triterpenoid saponins, and used in China as an over-the-counter drug for oral delivery to treat human liver diseases, such as acute and chronic hepatitis), with Sefsol^®^ 218 (oil), Cremophor^®^ EL and Labrasol^®^ (surfactants), and Transcutol^®^ P (cosurfactant) [113] or lutein, with Phosal^®^ 53 MCT (oil), Labrasol^®^, and Transcutol^®^ HP or Lutrol^®^ E400 (cosurfactants) [114]. To improve the dissolution, absorption and therapeutic efficacy of piroxicam (a nonsteroidal anti-inflammatory drug of the oxicam class used in the treatment of musculoskeletal, joint and other inflammatory disorders), self-nanoemulsifying DDS of its liquid and solid form was developed with different excipients *viz.* oils (liquid paraffin, ethyl oleate, coconut, maize, olive and soybean oils), surfactants and co-surfactants (Tween^®^ 20, 60, and 80; Labrasol^®^; Cremophor^®^ EL, and RH40; Transcutol^®^ HP; propylene glycol; poly(ethylene) 400). Liquid self-nanoemulsifying DDS composed of coconut or soybean oil, with Tween^®^ 80, Transcutol^®^ HP, was selected as the optimized formulation based on the solubility study and pseudo-ternary phase diagram. In vivo pharmacokinetic studies involving eight healthy human volunteers showed a significant improvement in the oral bioavailability of piroxicam from the solid supersaturatable preparation than both the pure drug and its commercial product (Feldene^®^) [115]. Self-nanoemulsions are also useful for improving the biological activity of some whole vegetable oils. Thus, to enhance the anti-inflammatory property of *Swietenia humilis* Z. (*S. humilis*, Pacific Coast mahogany) oil, self-nanoemulsifying systems involving this oil and three nonionic surfactants, i.e., Labrasol^®^, Tween^®^ 20, Capmul^®^, and Labrafil^®^, were developed. Nanoemulsions showed droplet size below 200 nm and low PDI (0.3). Carrageenan-induced rat paw oedema test exhibited that the anti-inflammatory effect of Pacific Coast mahogany oil was greater in the self-nanoemulsifying systems than alone [116].

#### 4.1.2. Nanoliposomes

By using the ethanol injection method, Charcosset et al. [117] developed Lipoid^®^ S100 (phospholipid obtained from soybean lecithin) liposomes loaded with the hydrophobic α-tocopherol, which was adopted as a model substance to be incorporated. Moreover, α-tocopherol-loaded liposomes remained stable up to 1 month at 5 °C, while the mean size and polydispersity index (PDI) ranged 89–112 nm and 0.138–0.181, respectively. With the same technique, Sebaaly et al. [118] encapsulated the EO of the flower of *Syzygium aromaticum* (*S. aromaticum*, clove) or eugenol (among the main constituents of clove EO conferring its medicinal and nutritional benefits) into Phospholipon^®^ 90H (phosphatidylcholine-based emulsifier) or Lipoid^®^ S100 liposomes. Homogenous (PDI < 0.2 by using Phospholipon^®^ 90H and <0.8 by using Lipoid^®^ S100), stable (for up 1 month at 4 °C), nanometric-sized (250–300 nm by using Phospholipon^®^ 90H and almost around 200 nm by using Lipoid^®^ S100) and multilamellar liposomes with a high phospholipid, eugenol loading rates and entrapment efficiency (EE) of clove EO components were obtained. These liposomes formulations are efficient to protect eugenol from UV light irradiation, maintaining the 2,2-diphenyl-1-picrylhydrazyl (DPPH) scavenging activity as an aqueous solution of free eugenol. To improve both the stability and delivery of rosemary EO exhibiting useful antioxidant and antimicrobial effects, encapsulating nanoliposomes were developed. The results showed that the liposomal EO had higher toxic effects on MCF7 cells due to enhanced drug delivery [119].

#### 4.1.3. Nanolipospheres

Lipospheres are a particulate dispersion containing solid spherical particles sized 0.2–100 μm, made of a solid hydrophobic fat core, consisting of TAGs or FAs derivatives, stabilized by a monolayer of phospholipids. Drugs are dissolved or dispersed in the internal solid fat matrix [120]. The neutral fats used in the preparation of the hydrophobic core include tricaprin, trilaurin, tristearin, stearic acid, ethyl stearate and hydrogenated vegetable oil [121].

Lipospheres represent a fat-based encapsulation system developed for parenteral and topical delivery of plant-derived bioactives, like demeric flavonoids, terpenoids, saponin, catechins and flavolignan, which increase the bioavailability following their encapsulation [122]. Lipid nanospheres have shown to be effective nanocarriers able to improve the oral bioavailability of tripterin (a plant-derived anticancer compound). Zhang et al. [123] prepared tripterin-loaded lipid nanospheres by the rapid dispersion of an ethanol mixture of tripterin, lecithin, sodium oleate, and soybean oil into the water. The obtained nanospheres were 150 nm in size, and stable, exhibiting a high value of EE (99.95%) and a negligible drug release (<0.2% in simulated physiological fluid). The pharmacokinetic analysis showed that the oral bioavailability of tripterin was enhanced by up to 224.88%. Mechanistic studies proved that this increased oral absorption was due to both improved intestinal permeation and post-enterocyte lymphatic transport.

#### 4.1.4. Nanostructured Lipid Carriers

Nanostructured lipid carriers (NLCs) belong to the youngest class of lipid-based nanocarriers, and over the last ten years, they have gained increasing interest. NLCs are composed of a mixture of solid and liquid lipids, which solubilizes the active pharmaceutical ingredient, stabilized by a surfactant. The lipid excipient miscibility and structural modifications (polymorphism) play an important role in formulation stability and are not easily predicted in the early pharmaceutical progress. Even if the excipients are macroscopically miscible, microscopic heterogeneity during storage will result in phase separation, which can only be observed after many months of stability studies [124]. As extensively detailed in Section 3.2.7, phytosterols are biosynthetically derived from squalene. Fang et al. [125] developed NLCs consisting of Precirol^®^ (glyceryl palmitostearate) and squalene for topical drug delivery. NLCs loading psoralen derivatives for psoriasis treatment were examined for their ability to permeate across the skin, as well as for their drug delivery efficiency. Enhanced permeation and controlled release of psoralen were both achieved using NLCs with squalene. The permeation of psoralens increased in the order of 8-methoxypsoralen > 5-methoxypsoralen > 4,5,8-trimethylpsoralen. Always, for a skin protection purpose, spherical and stable lipid nanocarriers, exhibiting self-antioxidative properties, minimal side effects and able to co-encapsulate and co-release synthetic filters against UVA and UVB rays (butyl-methoxydibenzoylmethane and octocrylene), were developed by using *Rubus ideaus* L. (*R. ideaus*, raspberry) and rice bran seed oils. Both vegetable oils led to a less ordered arrangement of the lipid core, that offers much space for the entrapment of large amounts of butyl-methoxydibenzoylmethane (79%) and octocrylene (90%), as wells as improving the antioxidant activity and UV absorption properties, particularly for the lipid nanocarriers prepared from rice bran oil. By formulating the lipid nanocarriers into creams containing only 3.5% of the UV filters and 10.5% of the vegetable oils, the resulting sunscreens exhibited improved photoprotection, reflecting up to 91% and 93% of UVA and UVB rays, respectively [126]. Zedoary turmeric oil is a traditional Chinese oily medicine isolate from *Curcuma zedoaria* R. that exerts positive health effect, such as protection against liver injury, tumor, and bacterial infections. In addition, it increases white blood cells and has antithrombotic activity. As a promising intravenous dosage form of water-insoluble oily drugs, zedoary turmeric oil-based NLCs were prepared by using Crodamol^®^ SS (solid lipid), Miglyol^®^ 812N (liquid oil), and soybean phosphatidylcholine (emulsifier). The blood concentration of the indexical component found in zedoary turmeric oil, germacrone, was assessed after the intravenous administration of these NLCs and compared with that of zedoary turmeric oil alone. Zedoary turmeric oil-based NLCs prolonged the acting time over the oil, just as in mice [127]. Lacatusu et al. [14] developed encapsulating nanocarriers made of a blend of *Amaranthus spp.* (amaranth) seeds oil and/or *Cannabis sativa* L. (*C. sativa*, hempseed) oil, able to improve both the bioavailability and the therapeutic benefit of lipophilic plant extract enriched in carotenoids like carotenoid extract originated from *Tagetes patula* L. (*T. patula*, Mexican marigold), which exerted a greater antioxidant activity as a result of their increased EE when carried into NLC. Recently, nanoscale oil bodies were spontaneously assembled from plant oils (olive, peanut, sesame, and soybean oils), phospholipids, and a protein obtained in *E. coli*, by fusing the anti-epidermal growth factor receptor affibody (Z_EGFR2_) with oleosin, a structural protein of plant seed oils inserted into the oil body TAG matrix to form a hairpin-like structure. Interestingly, this nanoformulation (around 200–300 nm) was (i) exclusively made of biomaterials and comprised a TAG core surrounded by a monolayer of phospholipids, with the acyl moieties of the phospholipids facing the TAG, and the phospholipids head group exposed to the cytosol, (ii) able to load the hydrophobic anticancer drug camptothecin, and (iii) selectively internalized by EGFR-positive lung cancer cells, with an efficiency exceeding 90%. As a result, a strong antitumor activity was found, also confirmed in vivo in tumor-bearing mice [128].

#### 4.1.5. Polymeric Nanoparticles

Due to their availability, biocompatibility, absence of toxicity and biodegradability, natural polymers like cellulose, chitosan, and alginate, are becoming very important to overcome issues restraining the pharmaceutical applications of phytochemicals, such as volatilization, water insolubility and thermolability and photodegradation. To resolve these problems, chitosan-alginate NPs were used for encapsulating vegetable oils rich in essential and non-essential FAs and other types of bioactive agents, such as *Curcuma longa* L. (*C. longa*, turmeric) [129] and, olive, *Plukenetia volubilis* L. (*P. volubilis*, sacha inchi), and soybean [130] oils. Notably, 2.48 nm sunflower seed oil-chitosan composite spheres as potential multifunctional drug carriers were synthesized by the encapsulation of sunflower seed oil in chitosan droplets. Interestingly, these spheres were able to encapsulate simultaneously either hydrophilic materials (i.e., iron oxide NPs that could be guided by a magnet) or lipophilic materials (i.e., rhodamine B or epirubicin) [131]. Ghaderi et al. [132] formulated stable 70–100 nm ethylcellulose NPs loading γ-oryzanol. Similarly, Liakos et al. [133] developed 95–185 nm cellulose acetate nanocapsules carrying *Cymbopogon citratus* (*C. citratus*, lemongrass) oil, able to adhere well to mucous membranes and to have very good antimicrobial properties at little concentrations against *E. coli* and *S. aureus*. Silibinine and pomegranate (*P. granatum*) oil have high therapeutic value owing to antioxidant activity, but their poor aqueous solubility restricts the biological efficiency of both bioactives. To overcome this issue, Marchiori et al. [134] developed nanocapsule suspensions with pomegranate oil (oil core) and ethylcellulose (polymeric wall) for silibinine encapsulation. Silibinine-loaded pomegranate oil-based nanocapsules showed an average diameter of 157 nm, homogenous size distribution, ζ-potential of −14.1 mV, pH of 5.6 and silibinine content close to 100%. For free silibinine, nanoencapsulation controlled better silibinine release and its scavenging capacity, without cytotoxicity against human monocytes and lymphocytes, in which minimal protein carbonyls and DNA damage were seen. Besides, lipid peroxidation occurred in nanocapsule treatments, regardless of the silibinine presence, which was attributed to pomegranate oil acting as a substrate in reaction. Interestingly, these nanocapsules exhibited anti-inflammatory effects on skin damage UVB radiation-induced in mice when embedded into gellan gum (a natural biomaterial product as microbial exopolysaccharide fermentation by *Pseudomonas elodea*) hydrogel [135]. As a strategy to overcome the limitations of antitumor, antioxidant, and anti-inflammatory 3,3′-diindolylmethane, Mattiazzi et al. [136] developed 3,3′-diindolylmethane-loaded nanocapsules composed by Eudragit^®^ RS100 or ethylcellulose (polymeric wall) and *Primula veris* L. (*P. veris*, primula) or *Prunus armeniaca* L. (*P. armeniaca*, apricot) oil (core). All formulations had nanometric size (around 190 nm), low PDI (<0.2), acid pH, high values of ζ-potential, drug content, and EE (about 100%). Nanoencapsulation protected 3,3′-diindolylmethane against UVC-induced degradation and increased the scavenging activity, while promoting a sustained release of the bioactive compound (in the range of 58–78% after 84 h) than its free form (86% after 12 h), as well as providing a superior cytotoxic effect against the human glioblastoma cell line (U87 cells) in the highest concentrations. Cirsiliol (5,3′,4′-trihydroxy-6,7-dimethoxyflavone) is an abundant bioactive plant flavonoid, which has been shown to exhibit inhibitory activity against phosphatidylinositol-3-kinase, an enzyme implicated in many cancer types. Despite its promising therapeutic benefits, cirsiliol has not yet been formulated into any type of dosage form. Thus, to enhance its biopharmaceutical properties, Al-Shalabi et al. [137] developed a polymeric nanoscale formulation for cirsiliol isolated from Jordanian *Teucrium polium* L. (*T. polium*, germander). Cirsiliol entrapped into core-shell NPs composed of castor oil-filled core and poly(ethylene glycol)-b-poly(ε-caprolactone), showing a mean diameter of 158.1 nm and an almost neutral surface charge, exhibited an EE of 53.5%, as well as a sustained drug release at pH 7.4, with 41% of cirsiliol released after four days. Cytotoxicity assays in MCF7 cells showed dose-dependent cytotoxicity of cirsiliol-NPs comparable to free cirsiliol.

#### 4.1.6. Solid Lipid Nanoparticles

*Melaleuca alternifolia* (*M. alternifolia*, tea tree) oil nanocapsules potentiate the treatment of trypanosomosis when associated with the trypanocidal drug diminazene aceturate, as shown in *Trypanosoma evansi*-infected mice [138]. Sesamol, a phenolic component of sesame seed oil, was packaged into solid lipid NPs to enhance its hepatoprotective bioactivity. These NPs, nearly spherical, and with an average particle size of 120.30 nm, showed significantly better hepatoprotection than free sesamol and a well-established hepatoprotective antioxidant silymarin in carbon tetrachloride-induced sub-chronic liver injury in rats [139]. Linalool is a highly volatile monoterpene alcohol (3,7-dimethyl-1,6-octadien-3-ol) found free or combined in the plant EOs, particularly lavender and coriander, showing anti-inflammatory, anticancer, anti-hyperlipidemic, antimicrobial, antinoceptive, analgesic, anxiolytic, antidepressant and neuroprotective properties [140]. High volatilization, poor solubility, then scanty bioavailability, of linalool has been overcome by loading it in NLCs. Linalool-NLCs had a size of 52.72 nm; the EE and drug loading gave 79.563 and 7.555%, respectively; the cumulative release of linalool from free linalool reached 51.414% at 180 min., while linalool from linalool-NLCs was 15.564%. Besides, the pharmacokinetics parameters, sustained-release effect and increased absorption of linalool-NLCs were better than those of linalool [141]. Interestingly, these NPs showed in vitro antiproliferative effects on hepatocarcinoma (HepG2) and lung adenocarcinoma (A549) cell lines in a dose-dependent response superior to free linalool [142].

### 4.2. Hybrid Nanoparticles

Aiming to protect and stabilize vegetable oils, hybrid organic NPs were synthesized by adding, one by one, oil of maize, hydrogenated and unhydrogenated castor, high-oleic sunflower, rapeseed oil, and soy to the imidization reaction of poly(styrene-maleic anhydride) with ammonium hydroxide in an aqueous environment [143]. Hybrid NPs based on polymer-lipids have been increasingly recognized as promising nanocarriers for lipophilic drugs [144]. Ghitman et al. [145] developed hybrid NPs based on poly(lactic-co-glycolic acid)-*Nigella sativa* L. (*N. sativa*, black cumin) oil, able to encapsulate and then to release lipophilic bioactives/drugs, such as 5-fluorouracil, α-tocopherol, curcumin, hydrocortisone, indomethacin, izohidrafural, nitrofurantoin, and resveratrol. As was expected, the selected vegetable oil, as a high lipophilic compound, determined an increase in the lipophilicity of the hybrid system, respectively a reduction of the solubility parameter. Liquid oil nanodroplets exhibited a protective effect upon the encapsulated lipophilic molecules. Except for hydrocortisone, nitrofurantoin and 5-fluorouracil, for which the EE decreased considerably, these NPs presented higher EE for almost all drugs, as compared to the standard polymeric matrix. The capacity of hybrid NPs to encapsulate all lipophilic drugs was at least double poly(lactic-co-glycolic acid) NPs.

### 4.3. Inorganic Nanoparticles

#### 4.3.1. Metallic Nanoparticles

As reported below, some metallic NPs have been used to strengthen the therapeutic effect of vegetable oils, while attenuating NP side effects. Cytotoxicity, wound healing, antioxidant and cell proliferation activities of the oil-in-water and water-in-oil emulsions obtained by combining the bioactive compounds black cumin, calendula extract and lipoic acid capped gold NPs, as assessed on Vero cells, showed an improved therapeutic effect over individually evaluated bioactives [146]. Similarly, biological silver NPs made by the saprophytic parasite fungus *Fusarium oxysporum* have proven a synergistic and additive effect with *Origanum vulgare* L. (*O. vulgare*, oregano) EO against multidrug-resistant bacteria, such as methicillin-resistant *S. aureus*, β-lactamase-, and carbapenemase-producing *E. coli* and *Acinetobacter baumannii* strains [147].

#### 4.3.2. Nanoclay Minerals

Low-density polyethylene/halloysite nanotubes are employed as efficient nanocarriers for carvacrol, a phenolic monoterpene present in the EOs of oregano and *Thymus vulgaris* L. (*T. vulgaris*, thyme). The resulting polymer nanocomposites exhibit outstanding antimicrobial properties, with inhibitory activity against *E. coli*, *Listeria innocua*, and *Alternaria alternate* [105].

## 5. Conclusions

Owing to their versatile association with enzymes, receptors and metabolic pathways, compounds and extracts obtained from natural sources continue to stand in the spotlight of drug design. Nanomedicine allows the efficient administration of natural products with improved bioavailability, targeting and controlled release, while preventing the alteration of active constituents following physicochemical changes. The interest of the scientific community in the field of nanosized delivery of natural compounds is demonstrated by the exponential growth of the publications in this field [148]. However, the applicability of plant extracts enriched in bioactives showing antioxidant, immunoregulatory, antimicrobial, and anticancer activities encounter several problems in the food and pharmaceutical sectors. Plant-derived molecules are either hydrophilic or lipophilic in nature. Highly hydrophilic bioactives have poor absorption via the lipid membrane which slows down their biological efficacy and pharmacokinetics [17]. Overall, issues concerning nutraceuticals relate to the composition, bioavailability, stability, weak oral absorption and GIT permeation, labile nature and target ability [13,14,15,16]. The use of certain vegetable oils in the pharmaceutical industry (e.g., garlic oil) is very limited, due to their lipophilic characteristics, volatilization, strong odour and low stability in gastrointestinal fluids, which reduces their bioavailability for systemic circulation, especially when provided orally [16]. The oral route is one of the most preferred drug administration ways for chronic therapy. The drug dissolution is a crucial step concerning the absorption processes of poorly water-soluble drugs. Drug dissolution is the most hindering step that occurs during the absorption of poorly water-soluble products. Approximately 40% of the marketed products are poorly soluble or lipophilic compounds, leading to reduced oral bioavailability [111]. Besides, the large molecular size of the plant bioactives also limited their therapeutic uses due to low membrane permeation [17]. Conversely, nanoformulations, by including nanometric delivering systems involving vegetable oil nutraceuticals, address most of these problems, offering several advantages, such as minimal carrier cytotoxicity, good storage stability, synergistic effects, antioxidant and sustained release, easy-to-scale-up production [14] and the ability to potentiate herbal bioactive efficacy by increasing solubility and absorption profile, while reducing the dosage and side effects [17]. Nanoformulations designed to improve the nutraceutical properties of vegetable oil-based compounds, as well as vegetable-oil-derived bioactives used to enhance the activity of the same drugs, are summarized in Table 1.

However, even though the use of nanotechnology is an alternative to improve the characteristics, aiming to ensure the stability and effectiveness of vegetable oils/vegetable oil-derived bioactives with pharmacological properties, further studies assessing the risk of the possible detrimental toxic effects of nanostructures are necessary [149].

## Figures and Tables

**Table 1:** Nanoformulations improving nutraceutical vegetable oil-based bioactives or vegetable-oil-derived bioactives enhancing drug activity ^1^.

Bioactive(s)	Issue(s) Solved ^2^	Nanocarrier ^3^	Ref. ^4^
α-tocopherol	Water solubility (hydrophobicity)	Phosphatidylcholine/soybean lecithin (Lipoid^®^ S100)-based liposome	[117]
α-tocopherol, benzylisothiocyanate	Release, stability (photodegradation) of curcumin	α-tocopherol/benzylisothiocyanate/polyoxyethylene sorbitan monooleate (Tween^®^ 80)/sodium stearoyl lactate-based oil-in-water nanoemulsion	[97]
Apricot (*P. armeniaca* L.) and primula (*P. veris* L.) oils	Bioactivity, release, stability (photodegradation) of 3,3′-diindolylmethane	Apricot or primula oil/ethylcellulose or ammonium methacrylate copolymer (Eudragit^®^ RS100)/polyoxyethylene sorbitan monooleate (Tween^®^ 80)/sorbitan monooleate (Span^®^ 80)-based nanocapsules loading 3,3′-diindolylmethane	[136]
Avocado (*P. americana*) oil	Bioavailability, stability	Ethoxylated lauryl ether (Ultrol^®^ L70)/keto-stearyl alcohol (Ultrol^®^ CE200)-based oil-in-water nanoemulsion loading octyl methoxycinnamate and solid particles of titanium dioxide	[99]
Bitter orange (*C. aurantium*) bloom EO	Bioactivity	Oil-in-water nanoemulsion	[110]
Black cumin (*N. sativa*) oil, calendula (*C**.** officinalis*) extract, gold NPs and lipoic acid	Bioactivity, side effects of bioactives	Black cumin oil/calendula extract/lecithin/ polyoxyethylene sorbitan trioleate (Tween^®^ 85)-based nanoemulsions enriched with lipoic acid capped gold NPs	[146]
Black cumin (*N. sativa*) oil	Bioavailability, release, water solubility (hydrophobicity) of 5-fluorouracil, α-tocopherol, curcumin, hydrocortisone, indomethacin, izohidrafural, nitrofurantoin, and resveratrol	Black cumin oil/poly(lactic-co-glycolic acid) hybrid NPs loading drug	[145]
Calendula (*C. officinalis*) infused black seed oil	Bioactivity	Lecithin/polyoxyethylene sorbitan monooleate (Tween^®^ 80)-based oil-in-water nanoemulsion	[100]
Camphor from rosemary (*R. officinalis*) EO	Bioactivity	Polyoxyethylene sorbitan monolaurate (Tween^®^ 20)-based oil-in-water nanoemulsion	[108]
Carotenoid-rich extract of pumpkin (*C. moschata*) pulp and seeds	Bioactivity	Butylated hydroxytoluene/polyoxyethylene sorbitan monooleate (Tween^®^ 80)/tetrahydrofuran-based oil-in-water nanoemulsion	[29]
Carotenoid-rich extract of Mexican marigold (*T. patula*)	Bioactivity, bioavailability, water solubility (hydrophobicity)	Blend of *Amaranthus spp.* (amaranth) seeds oil and/or hempseed (*C. sativa*) oil/cetyl palmitate/glycerol/ L-α-phosphatidylcholine/monostearate/polyoxyethylene sorbitan monolaurate (Tween^®^ 20)/poloxamer 188 (Synperonic^®^ PE/F68)-based NLC	[14]
Carvacrol from oregano (*O. vulgare)* and thyme (*T**.** vulgaris*) EOs	Bioactivity, stability (thermolability)	Low-density polyethylene/halloysite nanotube films loading carvacrol	[105]
Castor (*R. communis*) oil	Bioactivity, bioavailability, release of cirsiliol isolated from Jordanian germander (*T. polium*)	Castor oil/poly(ethylene glycol)-b-poly(ε-caprolactone)/polyoxyethylene sorbitan monooleate (Tween^®^ 80)/sorbitan monooleate (Span^®^ 80)-based nanocapsule loading cirsiliol	[137]
Chamber bitter (*P. urinaria*) ethanolic extract	Bioactivity, bioavailability, water solubility (hydrophobicity)	Polyoxyethylene sorbitan monooleate (Tween^®^ 80)/sorbitan monooleate (Span^®^ 80)-based self-emulsion	[112]
Ethyl oleate, coconut (*C. nucifera*), maize (*Z. mays*), olive (*O. europaea*), and soybean *(G. max*) oils	Absorption, bioactivity, water solubility (hydrophobicity) of piroxicam	Ethyl oleate, coconut, maize, olive, and soybean oils/poly(ethylene) glycol 400 or polyoxyethylene sorbitan monooleate (Tween^®^ 80)/diethylene glycol monoethyl ether (Transcutol^®^ HP)-based self-emulsion	[115]
Eugenol of/or clove (*S. aromaticum*) EO	Bioavailability, stability (photodegradation), water solubility (hydrophobicity)	Soybean lecithin (Lipoid^®^ S100)/soy phosphatidylcholine (Phospholipon^®^ 90H)-based liposome	[118]
γ-oryzanol	Stability, water solubility (hydrophobicity)	Ehylcellulose/γ-oryzanol/polyvinyl alcohol-based NPs	[132]
Garlic (*A. sativum*) oil blend comprising 30–50% diallyl disulfide, 10–13% diallyl trisulfide, and 5–13% allyl sulphide	Bioactivity	Polyoxyethylene sorbitan monooleate (Tween^®^ 80)-based oil-in-water nanoemulsion	[13,103]
Ginger (*A. zingiber*) EO	Bioactivity, water solubility (hydrophobicity) of mitomycin C	Ginger EO/polyoxyethylene sorbitan monooleate (Tween^®^ 80)/sorbitan monolaurate (Span^®^ 20)-based oil-in-water nanoemulsion	[109]
High-oleic sunflower (*H. annuus*), hydrogenated and unhydrogenated castor (*R. communis*), maize (*Z. mays*), rapeseed *(B. napus*) oils	Bioavailability, protection, stability	Imidized (with ammonium hydroxide in aqueous environment) poly(styrene-maleic anhydride)/oil hybrid NPs	[143]
Lecithin, sodium oleate, soybean *(G. max*) oil	Bioavailability, permeation, stability, water solubility (hydrophobicity) of tripterin	Lecithin/sodium oleate/soybean oil-based liposphere	[123]
Lemongrass (*C. citratus*) oil	Bioavailability, stability	Cellulose acetate/lemongrass oil-based nanocapsules	[133]
Linalool	Bioavailability, stability (high volatilization), water solubility (hydrophobicity)	Decanoyl/octanoyl-glycerides, glycerin monostearate, polyoxyethylene sorbitan monooleate (Tween^®^ 80), sorbitan monooleate (Span^®^ 80)-based NLCs loading linalool	[141]
	Bioactivity, bioavailability, release, stability (high volatilization), water solubility (hydrophobicity)	Cetyl esters (Crodamol^®^ SS), cetyl palmitate (Crodamol^®^ CP), myristyl myristate (Crodamol^®^ MM)-based NLCs loading linalool	[142]
Lutein	Bioavailability, water solubility (hydrophobicity)	Caprylcaproyl macrogol-8 glyceride (Labrasol**^®^**)/diethylene glycol monoethyl ether (Transcutol**^®^** HP)/phosphatidylcholine (Phosal**^®^** 53 MCT)/poly(ethylene) glycol 660 (Lutrol**^®^** E400)-based self-emulsion	[114]
Oleanolic acid	Bioavailability, water solubility (hydrophobicity)	Caprylcaproyl macrogol-8 glyceride (Labrasol**^®^**)/diethylene glycol monoethyl ether (Transcutol^®^ P)/macrogolglycerol ricinoleate (Cremophor^®^ EL)/propylene glycol caprylate (Sefsol^®^ 218)-based self-emulsion	[113]
Oleosin	Bioavailability, targeting, water solubility (hydrophobicity) of camptothecin	Olive (*O. europaea*), peanut (*A. hypogaea*), sesame (*S. indicum*), and soybean (*G. max*) oils/phospholipids/protein obtained in *E. coli* by fusing the anti-epidermal growth factor receptor affibody (Z_EGFR2_) with oleosin-based nanoscale oil body	[128]
Olive (*O. europaea*), sacha inchi (*P. volubilis*), soybean (*G. max*) oils	Biocompatibility, biodegradability, blood circulation time, mucoadhesiveness, toxicity, water solubility (hydrophobicity) of polymeric nanocarriers/lipophilic drugs	Alginate/chitosan/polyoxyethylene sorbitan monooleate (Tween^®^ 80)/olive, sacha inchi, soybean oils/poloxamer 407 (Pluronic^®^ F127)-based NP	[130]
Oregano (*O. vulgare)* EO, silver NPs	Strong organoleptic characteristics of oregano OE and resistance towards silver NPs	Silver NP produced by the saprophytic parasite fungus *Fusarium oxysporum* and oregano EO	[147]
Pacific Coast mahogany (*S. humilis*) oil	Bioactivity	Caprylcaproyl macrogol-8 glyceride (Labrasol**^®^**)/glycerol monooleate (Capmul^®^)/oleoyl macrogol-6 glycerides (Labrafil^®^ M1944CS)/polyoxyethylene sorbitan monolaurate (Tween^®^ 20)-based self-emulsion	[116]
Polyphenol-rich ethyl acetate fraction of pomegranate (*P. granatum*) seed oil	Bioactivity	Ethyl acetate/soy lecithin-based oil-in-water nanoemulsion	[102]
Pomegranate (*P. granatum*) oil and silibinine	Bioavailability, stability, water solubility (hydrophobicity)	Ethylcellulose, polyoxyethylene sorbitan monooleate (Tween^®^ 80), pomegranate oil, sorbitan monooleate (Span^®^ 80)-based nanocapsules loading silibinine	[134,135]
Quercetin	Stability, water solubility (hydrophobicity)	Acetone/castor oil/ethanol/phosphatidylcholine/poly(ethylene) glycol 660-12-hydroxystearate-based oil-in-water nanoemulsion	[98]
Raspberry (*R**.** ideaus*) and rice bran (*O. sativa*) seed oils	Bioactivity, bioavailability, stability, water solubility (hydrophobicity) of butyl-methoxydibenzoylmethane and octocrylene	Ceteareth 12 and 20, cetearyl alcohol, cetyl palmitate, glyceryl stearate (Emulgade^®^ SE/PF)/L-α-phosphatidylcholine/n-hexadecyl palmitate/polyoxyethylene sorbitan monolaurate (Tween^®^ 20)/poloxamer 188 (Synperonic^®^ PE/F68)/raspberry and rice bran seed oils-based NLC	[126]
Rosemary (*R. officinalis*) EO	Bioactivity, bioavailability, stability, water solubility (hydrophobicity)	Chloroform/cholesteryl hemisuccinate/L-α-phosphatidylethanolamine dioleoyl-based liposome	[119]
Sage (*S. officinalis*) EO	Bioactivity, bioavailability	Polyoxyethylene sorbitan monooleate (Tween^®^ 80)/sorbitan monooleate (Span^®^ 80)-based oil-in-water nanoemulsion	[107]
Sesamol from sesame (*S. indicum*) seed oil	Bioactivity, release, solubility (oxidation and photodegradation), water solubility (hydrophobicity)	Polyoxyethylene sorbitan monooleate (Tween^®^ 80)/soy lecithin-based solid lipid NP loading sesamol	[139]
Squalene	Bioavailability, permeation, water solubility (hydrophobicity) of psoralen derivatives (5-methoxypsoralen, 8-methoxypsoralen, 4,5,8-trimethylpsoralen)	Glyceryl palmitostearate (Precirol^®^)/hydrogenated soybean phosphatidylcholine/monoglycerides (Myverol^®^ 18-04 K)/polyoxyethylene sorbitan monooleate (Tween^®^ 80)/poloxamer 188 (Pluronic^®^ F68)/squalene-based NLC	[125]
Sunflower (*H. annuus*) seed oil	Encapsulation of both hydrophilic (i.e., iron oxide nanoparticles) and lipophilic (i.e., rhodamine B or epirubicin) materials	Acetic acid/chitosan/iron(II) chloride tetrahydrate/iron (III) chloride hexahydrate/ polyoxyethylene sorbitan monooleate (Tween^®^ 80)/sodium hydroxide/sunflower seed oil-based NP encapsulating epirubicin and/or iron oxide	[131]
Tea tree (*M. alternifolia*) oil	Bioactivity, bioavailability	Cetyl palmitate/polyoxyethylene sorbitan monooleate (Tween^®^ 80)/tea tree oil-based nanocapsules	[138]
Thymus (*T. daenensis*) EO	Bioactivity, bioavailability	Lecithin/polyoxyethylene sorbitan monooleate (Tween^®^ 80)-based oil-in-water nanoemulsion	[106]
Tocotrieniol-rich fraction of red PO (*C. renda*) oil	Bioactivity	D-α-tocopheryl polyethylene glycol 1000 succinate (vitamin E TPGS)/ethanol/glycerol/polyoxyl-15-hydroxystearate (Solutol^®^ HS-15)-based oil-in-water nanoemulsion	[101]
Turmeric (*C. longa*) oil	Bioavailability, stability, volatilization, water solubility (hydrophobicity)	Alginate/chitosan/polyoxyethylene sorbitan monooleate (Tween^®^ 80)-based NP	[129]
Zedoary turmeric (*C. zedoaria*) oil	Bioavailability, water solubility (hydrophobicity) of zedoary turmeric oil/lipophilic drugs	Caprylic/capric triglycerides (Miglyol^®^ 812N)/cetyl esters (Crodamol^®^ SS)/soybean phosphatidylcholine-based NLC	[127]

^1^ Listed in alphabetic order. ^2^ If not otherwise indicated, issue(s) is/are referred to bioactive(s) aside reported. ^3^ Some chemicals are vegetable oil-derived bioactive compounds; the plural of the specific nanocarrier (e.g., NPs and not NP) indicated that more than one formulation was developed/tested. ^4^ Reference(s). EO, essential oil; NLC, nanostructured lipid carrier; PO, palm oil.
